# Prevalence and correlates of common mental health problems and recent suicidal behaviour among female sex workers in Nairobi, Kenya: findings from the Maisha Fiti study

**DOI:** 10.1192/bjo.2021.637

**Published:** 2021-06-18

**Authors:** Alicja Beksinska, Zaina Jama, Rhoda Kabuti, Mary Kungu, Hellen Babu, Emily Nyakiri, Pooja Shah, Chrispo Nyabuto, Monica Okumu, Anne Mahero, Pauline Ngurukiri, Erastus Irungu, Wendy Adhiambo, Peter Muthoga, Rupert Kaul, Janet Seeley, Tara S Beattie, Joshua Kimani, Helen Weiss

**Affiliations:** 1 Department of Global Health and Development, London School of Hygiene and Tropical Medicine; 2 UK Partners for Health and Development in Africa (PHDA), University of Nairobi Institute of Tropical and Infectious Diseases; 3 University of Toronto; 4 MRC International Statistics & Epidemiology Group, Department of Infectious Disease Epidemiology, London School of Hygiene and Tropical Medicine

## Abstract

**Aims:**

Adverse childhood experiences (ACEs), poverty, violence and harmful alcohol/substance are associated with poor mental health outcomes in the general population. These risks are likely to be exacerbated among Female Sex Workers (FSWs), however there are few studies examining risks factors for mental health problems among FSWs. We examine the prevalence and correlates of common mental health problems including suicidal behaviour among FSWs in Kenya.

**Method:**

Maisha Fiti is a longitudinal study among FSWs randomly selected from Sex Worker Outreach Programme (SWOP) clinics across Nairobi. Baseline data were collected from June-December 2019. Mental health problems were assessed using the Patient Health Questionnaire (PHQ-9) for depression, the Generalised Anxiety Disorder tool (GAD-7) for anxiety, and the Harvard Trauma Questionnaire (HTQ-17) for Post-Traumatic Stress Disorder (PTSD). Recent suicidal behaviour was defined as reported suicide attempt or suicidal ideation in the past 30 days. Other measurement tools included the WHO Adverse Childhood Experiences (ACE) score, WHO Violence Against Women questionnaire, and the WHO ASSIST tool (to measure harmful alcohol/substance use in the past 3 months). Descriptive statistics and multivariable logistic regression were conducted in Stata 16.1.

**Result:**

Of 1039 eligible FSWs, 1003 FSWs took part in the study (response rate: 96%) with a mean age of 33.7 years. The prevalence of moderate/severe depression was 23.2% (95%CI: 20.7–25.9%), moderate/severe anxiety 11.0% (95%CI: 9.3–13.1%), PTSD 14.0% (95% CI: 12.2–16.5%) and recent suicidal behaviour 10.2% (95%CI: 8.5–12.2%) (2.6% suicide attempt; 10.0% suicidal ideation). Among women with any mental health problem 63.0% also had a harmful alcohol/substance use problem. One in four women (25%; 95%CI: 22.5–27.8%) had depression and/or anxiety and this was independently associated with higher ACE scores, hunger (skipped a meal in last week due to financial difficulties), death of a child, perceived sex work stigma and recent sexual/physical violence. PTSD was associated with higher ACE scores, hunger, increased STI prevalence (chlamydia trachomatis) and recent violence. Recent suicidal behaviour was associated with higher ACE scores, low literacy, hunger, and recent violence. Mental health problems and suicidal behaviour were less prevalent among women reporting social support.

**Conclusion:**

The high burden of mental problems among FSWs indicates a need for accessible services tailored for FSWs alongside broader structural interventions addressing poverty, harmful alcohol/substance use and violence. High rates of ACEs among this population indicates the need to consider early childhood and family interventions to prevent poor mental health outcomes.

Funding: Medical Research Council and the UK Department of International Development

